# Xuezhikang Capsule for Type 2 Diabetes with Hyperlipemia: A Systematic Review and Meta-Analysis of Randomized Clinical Trails

**DOI:** 10.1155/2015/468520

**Published:** 2015-07-13

**Authors:** Min Li, Qingyong He, Yinfeng Chen, Bo Li, Bo Feng, Zhenpeng Zhang, Jie Wang

**Affiliations:** ^1^Department of Cardiology, Guang'anmen Hospital, China Academy of Chinese Medical Sciences, Beixiange 5, Xicheng District, Beijing 100053, China; ^2^Beijing University of Chinese Medicine, Beijing 100029, China; ^3^Xiyuan Hospital, China Academy of Chinese Medical Sciences, Beijing 100091, China

## Abstract

*Objective*. To evaluate the efficacy and safety of Xuezhikang capsule treating type 2 diabetes with hyperlipidemia. *Methods*. We searched six databases to identify relevant studies published before January 2015. Two review authors independently extracted data and assessed the Cochrane risk of bias tool. We resolved disagreements with this assessment through discussion and a decision was achieved by consensus. *Results*. We included 21 studies (1548 participants). Treatment courses were at least 8 weeks. Overall, the risk of bias of included trials was unclear. Among them, 16 studies could conduct meta-analysis. The result showed that compared with routine group (5 studies), Xuezhikang group had more effect on decreasing TC, TG, LDL-C, and rising HDL-C. However, compared with statins group (11 studies), Xuezhikang group has less effect on decreasing TC, TG, and rising HDL-C. Meanwhile, two groups had no statistical differences of LDL-C level. *Conclusion*. Xuezhikang capsule may be effective for treating type 2 diabetes with hyperlipemia. Our findings should be considered cautiously due to unclear risk of bias of the included studies and low methodological quality. Therefore, more strictly designed large-scale randomized clinical trials are needed to evaluate the efficacy of Xuezhikang capsule in type 2 diabetes with hyperlipemia.

## 1. Background

In recent years, with the improvement of people's living standard, lifestyle changes, and the aging of the population, the incidence of type 2 diabetes has become very high with an obvious rising trend. It has been a threat to global human health and become one of the most important public health problems worldwide [1]. Because of the disorder of biological regulation function of insulin, diabetes patients are often accompanied by lipid metabolism disorders, complicated with hyperlipidemia [2]. Diabetes complicated with hyperlipidemia can accelerate the process of atherosclerosis, increasing the incidence of cardiovascular disease [3]. At present, clinical nondrug therapy to treat diabetic with hyperlipidemia include diet, losing weight, exercise, and smoking and alcohol limit. Drugs to treat diabetic patients mainly regulate lipid metabolism such as statins and fenofibrate; these drugs can lower total cholesterol (TC) and low-density lipoprotein cholesterol (LDL-C) and are widely used in clinic [4]. Statins, however, can cause gastrointestinal disorders, rash, muscle tenderness, transaminase and creatine kinase eleations, rhabdomyolysis, and many other adverse reactions, so the application is limited [5]. Therefore, seeking new safe and effective lipid-lowering drugs is a hot spot of current research. Red kojic rice is a kind of traditional Chinese medicine which can be used as medicine or as food diet. It can invigorate spleen to promote digestion, clear damp, eliminate phlegm, and promote blood circulation to remove blood stasis. The components of Xuezhikang capsule (hereinafter referred to as XZK) is derived from red kojic rice by high tech biotechnology, containing lovastatin and statin homologue and a variety of essential amino acids, unsaturated fatty acid, sterol, and small amounts of flavonoids. It has obvious effects on dyslipidemia and atherosclerosis and has less adverse reaction [6]. Therefore, in the treatment of type 2 diabetes with hyperlipidemia, XZK may have good prospect of development.

## 2. Objective

Our objective is to evaluate the efficacy and safety of XZK treating type 2 diabetes with hyperlipidemia.

## 3. Methods

### 3.1. Criteria for Considering Studies for This Review

#### 3.1.1. Types of Studies

Randomized controlled trials with blind method or not and no limit of publishing language are included. We planned to include randomized crossover trials, if available. We excluded quasirandomized controlled trials (quasi-RCTs) and nonrandomized controlled trials.

#### 3.1.2. Types of Participants

Patients diagnosed with type 2 diabetes mellitus and hyperlipidemia were included, not including those with severe heart disease and serious complications. Age and race are not limited.

#### 3.1.3. Diagnostic Criteria

Diagnostic criteria for type 2 diabetes mellitus are as follows: the WHO diabetes diagnostic criteria suggested by diabetes expert advisory committee; 2005 American diabetes clinical guidelines (ADA) and the Adult Treatment Panel III (ATP III) of American cholesterol education program; American Diabetes Association criteria; 2010 China diabetes prevention and treatment guidelines. Diagnostic criteria for hyperlipemia are as follows: dyslipidemia prevention advice of china, 1997; 2007 Chinese adult dyslipidemia prevention guide standards; disease clinical diagnosis: criteria of cure and amelioration; guiding principle of traditional Chinese medicine clinical research. Randomized controlled trials that meet both diagnostic criteria are included.

#### 3.1.4. Types of Interventions

Treatment groups are given XZK and basic glucose-lowering drugs (hereinafter referred to as BGLD). Control groups are given placebos, statins, fenofibrates, and BGLD. Treatment courses are at least 8 weeks. There is no difference between treatment groups and control groups in gender, age, blood lipid, and blood glucose before treatment in every study.

#### 3.1.5. Types of Outcome Measures

Validity is calculated as follows: by comparison of patients' total cholesterol (TC), triglyceride (TG), high density lipoprotein cholesterol (HDL-C), and low density lipoprotein cholesterol (LDL-C) before and after treatment. Safety is calculated as follows: first, laboratory examination is as follows: peripheral hemogram, liver function, renal function, and so forth; second, adverse event is as follows: gastrointestinal reaction, allergic reaction, and so forth.

#### 3.1.6. Effective Criteria

Effective criteria are as follows: guiding principle of traditional Chinese medicine clinical research by Ministry of Health, 1993; cardiovascular drug clinical research guiding principles by Ministry of Health, 1998; advice of cardiovascular drug clinical trial evaluation method. The three evaluation standards agreed: remarkable effective: TC decreased by 20% or more, TG decreased by 40% or more, and HDL-C rose 0.26 mmol/L or more; effective: TC decreased by 10%~20%, TG decreased by 20%~40%, and HDL-C rose 0.10~0.26 mmol/L; noneffective: those which did not reach the effective criteria, TC rose by 10% or more, TG rose by 10% or more, and HDL-C decreased more than 0.18 mmol/L. The total effective rate = remarkable effective rate + effective rate.

### 3.2. Search Methods for Identification of Studies

The authors searched PubMed, The Cochrane Library, Chinese BioMedical Database (CBM), China National Knowledge Infrastructure (CNKI), Wanfang Database, and Chinese Scientific Journal Database (VIP) (publishing time was before January 2015). The following search terms were used individually or combined: “xuezhikang”, “type 2 diabetes mellitus”, “diabetes mellitus”, “hyperlipemia”, “HLP”, “hypercholesterolemia”, “hipertrigliceridemia”, “high-density lipoproteincholesterol”, “low-density lipoprotein cholesterol”, “mixed dyslipidemia”, “Total Cholesterol”, “TC”, “triglyceride”, “TG”, “HDL-C”, “LDL-C”, “dyslipidemia”, “lipid metabolism disorders”, and “hypercholesterolemia”. Further details were given in the following part. After removing duplicates from different databases, the authors confirmed RCTs by reading title, abstract, and even the full article.

*Search Strategies*

*Search Terms*

*Pubmed (Search in All Fields)*. Consider the following:

#1 Search (((((((((((((((HLP) OR hipertrigliceridemia) OR hypercholesterolemia) OR high-density lipoproteincholesterol) OR low-density lipoprotein cholesterol) OR mixed dyslipidemia) OR total cholesterol) OR TC) OR triglyceride) OR TG) OR HDL-C) OR LDL-C) OR dyslipidemia) OR hyperlipemia) OR lipid metabolism disorders) OR hypercholesterolemia.
#2 Search (xuezhikang).
#3 Search (type 2 diabetes) OR diabetes.
#4 (#1 and #2 and #3).

*The Cochrane Library (Search in All Fields)*. Consider the following:

#1 MeSH descriptor lipid metabolism disorders explode all trees.
#2 (HLP or hipertrigliceridemia or hypercholesterolemia or high-density lipoproteincholesterol or low-density lipoprotein cholesterol or mixed dyslipidemia or total cholesterol or TC or triglyceride or TG or HDL-C or LDL-C or dyslipidemia or hyperlipemia or hypercholesterolemia).
#3 (#1 or #2).
#4 (xuezhikang).
#5 (type 2 diabetes or diabetes).
#6 (#3 and #4 and #5).

*China National Knowledge Infrastructure (CNKI)*. Consider the following:

 (SU = “xuezhikang”) AND (SU = “type 2 diabetes” OR SU = “diabetes”) AND (SU = “lipid metabolism disorders” OR SU = “HLP” OR SU = “hipertrigliceridemia” OR SU = “hypercholesterolemia” OR SU = “high-density lipoproteincholesterol” OR SU = “low-density lipoprotein cholesterol” OR SU = “mixed dyslipidemia” OR SU = “total cholesterol” OR SU = “TC” OR SU = “triglyceride” OR SU = “TG” OR SU = “HDL-C” OR SU = “LDL-C” OR SU = “dyslipidemia” OR SU = “hyperlipemia” OR SU = “hypercholesterolemia”).

*Chinese Scientific Journal Database (VIP)*. Consider the following:

 (m = (lipid metabolism disorders + HLP + hipertrigliceridemia + hypercholesterolemia + high-density lipoproteincholesterol + low-density lipoprotein cholesterol + mixed dyslipidemia + total cholesterol + TC + triglyceride + TG + HDL-C + LDL-C + dyslipidemia + hyperlipemia + hypercholesterolemia)) *∗* (m = (type 2 diabetes + diabetes)) *∗* (m = xuezhikang).

*WanFang Database*. Consider the following:

 (Theme = (lipid metabolism disorders + HLP + hipertrigliceridemia + hypercholesterolemia + high-density lipoproteincholesterol + low-density lipoprotein cholesterol + mixed dyslipidemia + total cholesterol + TC + triglyceride + TG + HDL-C + LDL-C + dyslipidemia + hyperlipemia + hypercholesterolemia)) *∗* (Theme = (type 2 diabetes + diabetes)) *∗* (Theme = xuezhikang).

*Chinese BioMedical Database (CBM)*. Consider the following:

 (Theme = xuezhikang) AND (Theme = (type 2 diabetes OR diabetes)) AND ((Theme = (lipid metabolism disorders OR HLP OR hipertrigliceridemia OR hypercholesterolemia OR high-density lipoproteincholesterol OR low-density lipoprotein cholesterol OR mixed dyslipidemia OR total cholesterol OR TC OR triglyceride OR TG OR HDL-C OR LDL-C OR dyslipidemia OR hyperlipemia OR hypercholesterolemia))).

Note the following: “SU” is “Theme”; “m” is “title” and “keywords”; “*∗*” is “and”; “+” is “or”.

### 3.3. Data Extraction and Management

#### 3.3.1. Data Extraction and Management

Two authors conducted the literature searching, study selection, and data extraction independently. The extracted data of included studies was filled in form designed beforehand. The extracted data include age, sex, study size, details of treatment process, details of the control interventions, outcomes, and adverse effects. Two authors discussed settlement when in disagreement

#### 3.3.2. Unit of Analysis Issues

Subgroups were divided according to course of treatment and analyzed individually.

#### 3.3.3. Assessment of Risk of Bias in Included Studies

According to Cochrane Reviewer's Handbook 5.3, 6 evaluation criteria of the quality of randomized controlled trials were used, which include the generation of random sequence, randomization concealment, blind method, integrity of outcome data, selective reporting, and other bias.

#### 3.3.4. Measures of Treatment Effect

Revman 5.2 software provided by the Cochrane Collaboration was used for data analyses. Continuous outcomes were presented as weighted mean difference (WMD), dichotomous data as risk ratio (RR) and 95% confidence interval (CI).

#### 3.3.5. Assessment of Heterogeneity

Clinical heterogeneity of included studies was analyzed with *χ* ^2^ test. If *I* ^2^ < 50%, then there is no statistical heterogeneity between studies, and fixed effect model was used for data analysis; if *I* ^2^ > 50%, then statistical heterogeneity between studies exists, random effect model was used, and the cause of heterogeneity was analyzed. Subgroup analysis was used when clinical heterogeneity exists. Subgroup was divided according to the sources of heterogeneity, such as age, course, and dosage of medicines. Descriptive analysis was used if clinical heterogeneity still exists.

#### 3.3.6. Sensitivity Analysis

Compare the pooled statistics before and after excluding studies of low quality and great weight and those which have different result from other studies. If they have the same result (both results have differences or have no differences), then the meta-analysis result is stable and vice versa.

## 4. Results

### 4.1. Search Process

**Figure 1:**
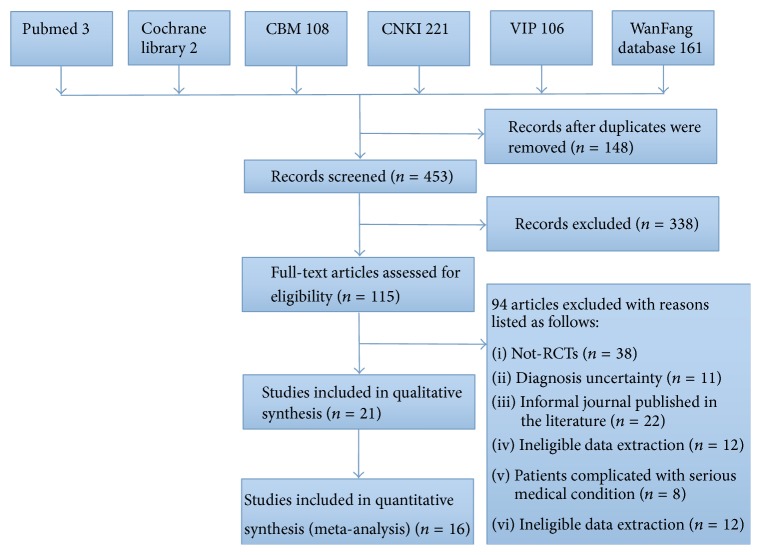
The flow chart of randomized clinical trials selection process.

The initial search using the electronic search strategies yielded 601 studies. After removing 148 duplicates from different databases, we kept 453 potentially relevant articles for further assessment. After reading the title and abstract, we excluded 338 studies. By further investigation of the full articles, we excluded 94 studies. Eventually we included 21 studies [7–25] for qualitative synthesis; among them there are 16 studies on which meta-analysis could be conducted [7–23]. A flow chart (Figure 1) showed the search process and study selection.

### 4.2. Included Studies

We included 21 studies in this review. Further details were given in Table 1. All studies were conducted in China and published in full. We did not identify any unpublished studies. The research [9] had two different control groups, two results were analyzed; the research [21] had two different times of therapy, so results were analyzed twice.

### 4.3. Participants

A total of 1548 participants with type 2 diabetes mellitus and hyperlipidemia were included in the 21 studies. The average sample size of the trials was 74 participants (ranging from 43 to 268 participants per trial). The age of the participants ranged from 40 to 85 years old.

### 4.4. Interventions

There were 11 studies for XZK plus BGLD versus statins plus BGLD [7–17]; 5 studies for XZK plus BGLD versus BGLD [18–21]; 3 studies for XZK plus BGLD versus placebo plus BGLD [22–24]; one study for XZK plus BGLD versus fenofibrate plus BGLD [9]; one study for XZK plus BGLD versus Duoxikang capsule plus BGLD [25].

### 4.5. Outcomes

There were 18 studies [7–21, 25] which were continuous variables. Among them, 17 studies reported blood lipid parameter, such as TC, TG, HDL-C, and LDL-C. Only one study [18] did not incorporate the LDL-C as observation index. There were 3 studies [22–24] which were dichotomous variables (set a threshold, reach the standard of the threshold for effective, whereas as invalid). Among them, 3 studies reported TC, TG, and HDL-C. The results were shown in Tables 2 and 3.

### 4.6. Risk of Bias Assessment

**Figure 2:**
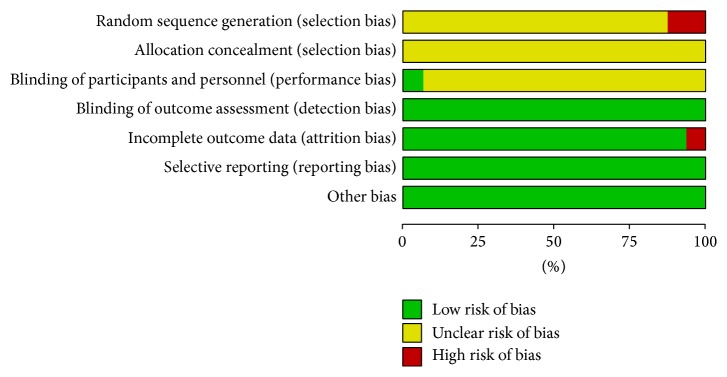
Methodological quality graph: review authors' judgements about each methodological quality item presented as percentages across all included studies.

**Figure 3:**
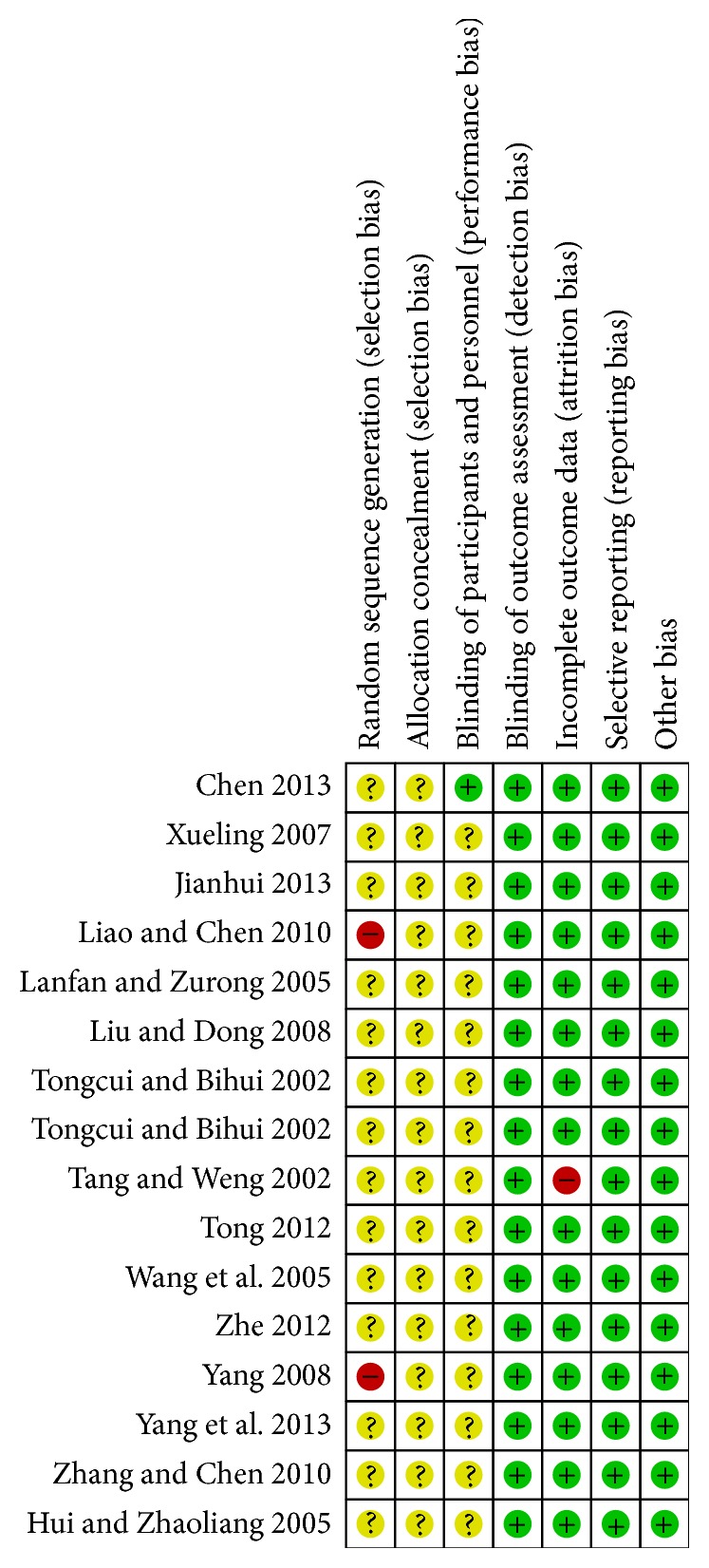
Methodological quality summary: review authors' judgements about each methodological quality item for each included study.

All the trials provided very limited information about design and methodology. The included studies did not describe allocation concealment clearly; only two studies [7, 15] chose patients according to the different treatment. One study [14] reported blinding of participants and personnel; other studies did not mention it. Four studies [18, 22–24] had incomplete outcome data, which did not refer to LDL-C. None of the trials had a pretrial estimation of sample size, which indicated the lack of statistical power to ensure appropriate estimation of the therapeutic effect, as shown in Figures 2 and 3.

### 4.7. Effects of Interventions

Due to clinical heterogeneity, only 16 studies [17, 19] could conduct meta-analysis. There were 11 studies [7–17] for XZK plus BGLD versus statins plus BGLD, and they were all continuous variables; there were 5 studies [18–21] for XZK plus BGLD versus BGLD, and they were all continuous variables; there were 2 studies [22, 23] for XZK plus BGLD versus placebo plus BGLD, and they were all dichotomous variables. Due to the number of dichotomous variables being a little less, so the authors did not conduct meta-analysis for dichotomous variables.

#### 4.7.1. Effects of Reducing TC

**Figure 4:**
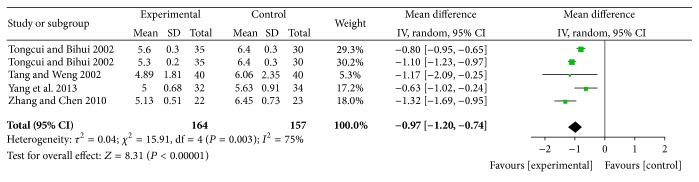
XZK plus BGLD versus BGLD (TC).

**Figure 5:**
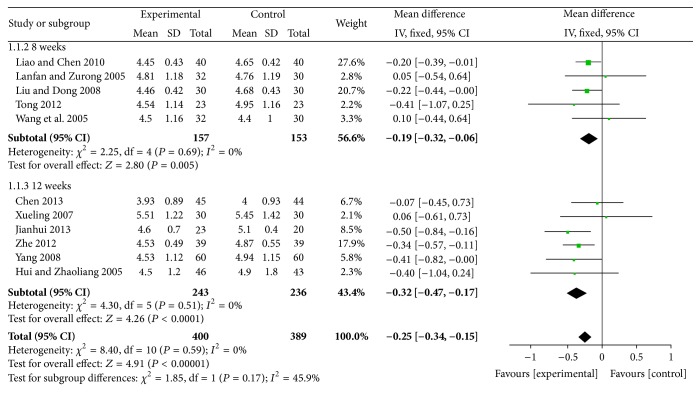
XZK plus BGLD versus statins plus BGLD (TC).

The results of reducing TC were statistically significant for XZK plus BGLD versus BGLD (WMD = −0.97, 95% CI: −1.2~−0.74, and *P* < 0.01), which reflected that XZK had definite effects on reducing TC. And the results of reducing TC were also statistically significant for XZK plus BGLD versus statins plus BGLD (WMD = −0.25, 95% CI: −0.34~−0.15, and *P* < 0.01), which reflected that XZK was better than statins on reducing TC, as shown in Figures 4 and 5.

#### 4.7.2. Effects of Reducing TG

**Figure 6:**
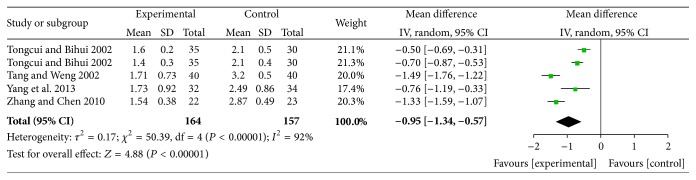
XZK plus BGLD versus BGLD (TG).

**Figure 7:**
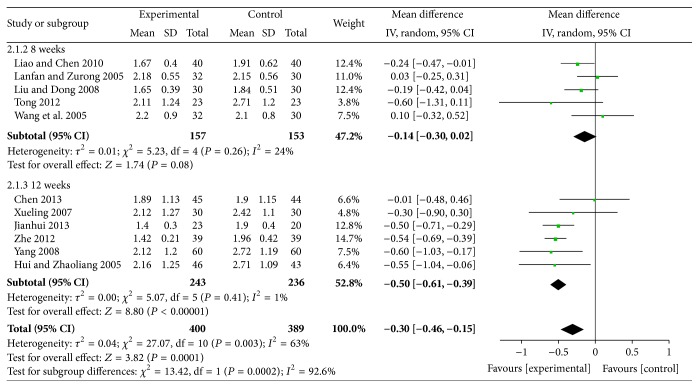
XZK plus BGLD versus statins plus BGLD (TG).

The results of reducing TG for XZK plus BGLD versus BGLD were WMD = −0.95, 95% CI: −1.34~−0.57, and *P* < 0.01. The results were statistically significant. Those results showed that XZK is effective in reducing TG. As for XZK plus BGLD versus statins plus BGLD, WMD = −0.3, 95% CI: −0.46~−0.15, and *P* < 0.01, which reflected that XZK was better than statins in reducing TG, as shown in Figures 6 and 7.

#### 4.7.3. Effects of Increasing HDL-C

**Figure 8:**
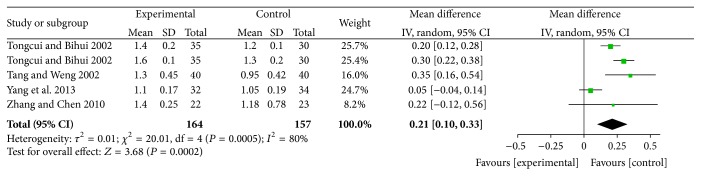
XZK plus BGLD versus BGLD (HDL-C).

**Figure 9:**
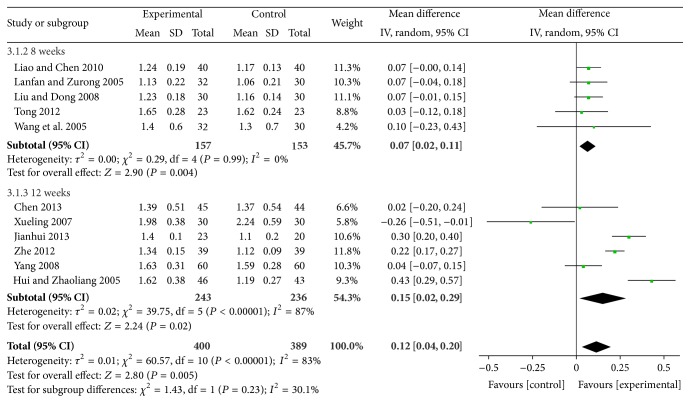
XZK plus BGLD versus statins plus BGLD (HDL-C).

For increasing HDL-C, XZK plus BGLD versus BGLD was WMD = 0.21, 95% CI: 0.1~0.33, and *P* < 0.01, which was statistically significant, so XZK has effects on increasing HDL-C. The results for XZK plus BGLD versus statins plus BGLD were also statistically significant (WMD = 0.12, 95% CI: 0.04~0.2, and *P* < 0.01), which reflected that XZK was better than statins to increase HDL-C, as shown in Figures 8 and 9.

#### 4.7.4. Effects of Reducing LDL-C

**Figure 10:**
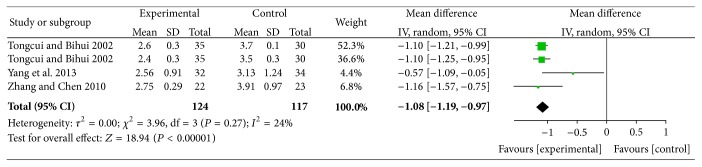
XZK plus BGLD versus BGLD (LDL-C).

*(1) XZK Plus BGLD versus BGLD*. On reducing LDL-C, the results were statistically significant for XZK plus BGLD versus BGLD (WMD = −1.08, 95% CI: −1.19~−0.97, and *P* < 0.01), which reflected that XZK had a good effect on reducing LDL-C, as shown in Figure 10.

**Figure 11:**
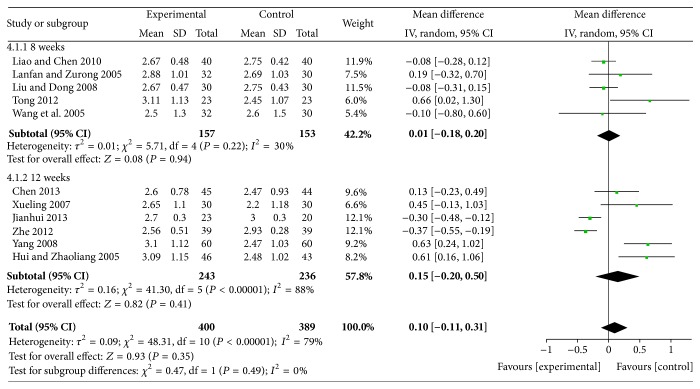
XZK plus BGLD versus statins plus BGLD (LDL-C).

*(2) XZK Plus BGLD versus Statins Plus BGLD*. The results for XZK plus BGLD versus statins plus BGLD on reducing LDL-C were not statistically significant (WMD = 0.1, 95% CI: −0.11~0.31, and *P* > 0.05), which means that maybe XZK is similarly effective compared to statins to reduce LDL-C, as shown in Figure 11.

According to different course of treatment, studies of XZK plus BGLD versus statins plus BGLD could be divided into 2 subgroups: 8 weeks and 12 weeks. There were 5 studies with course of treatment of 8 weeks; the results were (1) effects of reducing TC, WMD = −0.19, 95% CI: −0.32~−0.06, *P* < 0.01 (statistically significant), (2) effects of reducing TG, WMD = −0.14, 95% CI: −0.3~0.02, and *P* > 0.05 (not statistically significant), (3) effects of reducing LDL-C, WMD = 0.01, 95% CI: −0.18~0.2, and *P* > 0.05 (not statistically significant), and (4) effects of increasing HDL-C, WMD = 0.07, 95% CI: 0.02~0.11, and *P* < 0.016 (statistically significant). There were 6 studies with course of treatment of 12 weeks: (1) effects of reducing TC, WMD = −0.32, 95% CI: −0.47~−0.17, and *P* < 0.01 (statistically significant), (2) effects of reducing TG, WMD = −0.3, 95% CI: −0.46~−0.15, and *P* < 0.01 (statistically significant), (3) effects of reducing LDL-C, WMD = 0.15, 95% CI: 0.02~0.29, and *P* < 0.01 (statistically significant), and (4) effects of increasing HDL-C, WMD = 0.15, 95% CI: −0.2~0.5, and *P* > 0.05 (not statistically significant).

### 4.8. Adverse Effect

Except for 4 studies [10, 16, 17, 20], all the studies included had reported adverse effects. 794 patients in 21 studies took XZK. 7 patients, reported in 6 studies [7, 8, 12, 13, 19, 25] in total, had gastrointestinal symptoms, including abdominal distension and constipation. 2 study [9, 25] reported 3 cases of nausea and 1 [19] of rash. The adverse effects rate was 1.39%. 391 cases of patients in 11 studies took statins. 3 studies [12–14] reported that 14 patient had abdominal distension and constipation, 3 [14] had abdominal pain, and 1 [13] had elevated ALT levels. 4 study [7–9, 13] reported 8 cases of nausea. The adverse effects rate was 6.65%. 30 cases of patients took fenofibrate in 1 study [9], in which 3 had nausea and 5 had abdominal distension and anorexia. The adverse effects rate was 26.67%. All the above indicate that XZK had less adverse effects than statins and fenofibrate. However, adverse effects in source materials were not statistically analyzed, so the safety of XZK, statins, and fenofibrate remained to be studied in clinical trials of the future.

## 5. Discussion

### 5.1. Effectiveness of XZK

HMG-CoA reductase is the key enzyme for cholesterol synthesis. Statins block hydroxyl pentanoic acid metabolic pathways in cells by its competitive inhibition toward HMG-CoA reductase and thus block the synthesis of cholesterol, reducing blood cholesterol levels [26]. General statins can lower TC and LDL-C, slightly elevate HDL-C, and slightly lower TG. The main ingredients of XZK are lovastatin and statin homologue; therefore, XZK should have similar effects. This study followed the Cochrane principle of system evaluation and analyzed 16 studies of XZK treating diabetes mellitus with hyperlipidemia. The result shows that, compared with routine group, XZK group has more significant TC, TG decrease, and HDL-C rise. However, compared with statins group, XZK group has less effect on TC, TG decrease, and HDL-C rise, as well as no statistical differences of LDL-C level. The reasons might be as follows.

#### 5.1.1. The Effects of XZK towards Insulin Resistance and Abnormal Glucose Regulation

For diabetic patients, due to low insulin level or insulin resistance, the combining capacity of insulin and insulin receptor is deficient and the effects after combining are limited. Utilization and processing of glucose as well as glucose uptake capacity of muscle and adipose tissue are decreased, hepatic glucose production is increased, and activity of lipoprotein lipase decreased, causing glucolipid metabolic disorders [27]. XZK not only can regulate blood fat, but also has certain effect on the improvement of the insulin resistance. Wu compared XZK plus BGLD versus BGLD, to treat diabetic hyperlipemia; the result shows that XZK can significantly improve the insulin sensitive index (*P* < 0.01), while the basic treatment group has no significant change, which indicates that XZK can treat insulin resistance in type 2 diabetes patients [21]. Moreover, XZK is extracted from red yeast rice, a Chinese herbal medicine which has effects of invigorating spleen and promoting digestion, expelling phlegm and damp, promoting blood circulation, and removing blood stasis. In traditional Chinese medicine, diabetes is called xiaoke, in which, due to long-time diet disorder, overconsumption of fat and sweet food damages the spleen. Thus the transportation and transformation functions of spleen are frustrated, causing inner heat to dry the fluid of the body and eventually causing xiaoke [28]. As for hyperlipidaemia, in traditional Chinese medicine it is considered because of dysfunction of the spleen in transport, blood stasis, and phlegm-damp. XZK can invigorate spleen to promote digestion, expel phlegm and damp, promote blood circulation, and remove blood stasis [29]. It can adjust both blood sugar and blood fat which makes it a good choice for diabetic hyperlipemia patients.

#### 5.1.2. Limitation of This Study

The studies included have some limit, such as small size of case and low quality of methodology. Therefore, large-scale prospective, double-blinded controlled clinical trials are needed to confirm the validity and safety of XZK, especially to see whether it is safe and effective for statin intolerance patients. And, among these studies, the same evaluation index of different studies found different method of measurement. Some use measurement data but others use enumeration data which cause lots of information. In addition, the studies included basically studied the influence of drugs on blood lipids. Observation of endpoint criteria, such as occurrence of cardiovascular and cerebrovascular events, and long-term follow-up and safety observation can make the evidence stronger. And the patients of the studies included are mainly Chinese and the diagnostic criteria are also Chinese criteria. International criteria should be used to cover more patients of various regions thus expanding application range of XZK. Due to insufficient number of trials, we failed to perform funnel plot to detect publication bias. The limit above could influence the result to some extent, so the result of the study on the effects is relatively not so reliable.

### 5.2. Safety of XZK

Combined hyperlipidemia is common in patients with diabetes, and lipid metabolism disorder is an important cause of atherosclerosis and microangiopathy, involving the corresponding tissues and organs which cause coronary heart disease, hypertension, myocardial infarction, stroke, and so forth. Therefore, for patients with type 2 diabetes mellitus and hyperlipidemia, safe and effective lipid-regulating drugs are very necessary. Compared with XZK, statins have more adverse reactions such as gastrointestinal disorders, rash, transaminase and creatine kinase eleations, and rhabdomyolysis. Adverse reactions of short-term course of XZK are mainly gastrointestinal reaction and barely abnormal function of liver and muscle pain. Safety of long-term course of XZK remains to be seen.

### 5.3. Dose-Effect Relationship

There are not enough studies involved dose-effect relationship between dose of XZK and its effects, so this systematic review did not analyze dose-effect relationship.

### 5.4. Reporting Biases

There are not enough studies included in each subgroup, so this systematic review did not analyze reporting biases.

### 5.5. Practicability and Clinical Significance of This Study

The analysis of included studies shows that XZK may be effective for treating type 2 diabetes with hyperlipemia. Compared with other blood lipid regulating drug, XZK has better cost-effectiveness because of its favorable price. Nevertheless, through this systematic review, we found some points to suggest that clinical trials in the future should pay attention. First, choose adequate random methods and hide the dividing project to assure comparability and reduce selective bias. Double blind or triple blinding should be used. Second, the diagnostic criteria and effect evaluation criteria should follow international standard. Third, observed indexes should also contain occurrence rate of cardiovascular and cerebrovascular events. Fourth, clinical trial overseas should be carried out to observe individual difference between regions.

## 6. Conclusion

Xuezhikang capsule may be effective for treating type 2 diabetes with hyperlipemia. Our findings should be considered cautiously due to unclear risk of bias of the included studies and low methodological quality. Therefore, more strictly designed large-scale randomized clinical trials are needed to evaluate the efficacy of Xuezhikang capsule in type 2 diabetes with hyperlipemia.

## Figures and Tables

**Table 1:** The basic information of each study.

Study ID	Sample (T/C)	Intervention group	Control group	Course (week)	Outcome measures
Liao and Chen 2010 [7]	40/40	XZK + BGLD	Simvastatin + BGLD	8	TC, TG, HDL-C, LDL-C
Liu and Dong 2008 [8]	30/30	XZK + BGLD	Simvastatin + BGLD	8	TC, TG, HDL-C, LDL-C
Wang et al. 2005 [9]	32/30	XZK + BGLD	Pravastatin + BGLD	8	TC, TG, HDL-C, LDL-C
	32/30	XZK + BGLD	Fenofibrate + BGLD	8	TC, TG, HDL-C, LDL-C
Lanfan and Zurong 2005 [10]	32/30	XZK + BGLD	Simvastatin + BGLD	8	TC, TG, HDL-C, LDL-C
Tong 2012 [11]	23/23	XZK + BGLD	Simvastatin + BGLD	8	TC, TG, HDL-C, LDL-C
Zhe 2012 [12]	39/39	XZK + BGLD	Atorvastatin + BGLD	12	TC, TG, HDL-C, LDL-C
Xueling 2007 [13]	30/30	XZK + BGLD	Fluvastatin + BGLD	12	TC, TG, HDL-C, LDL-C
Chen 2013 [14]	45/44	XZK + BGLD	Simvastatin + BGLD	12	TC, TG, HDL-C, LDL-C
Yang 2008 [15]	60/60	XZK + BGLD	Simvastatin + BGLD	12	TC, TG, HDL-C, LDL-C
Jianhui 2013 [16]	23/20	XZK + BGLD	Rosuvastatin + BGLD	12	TC, TG, HDL-C, LDL-C
Hui and Zhaoliang 2005 [17]	46/43	XZK + BGLD	Simvastatin + BGLD	12	TC, TG, HDL-C, LDL-C
Tang and Weng 2002 [18]	40/40	XZK + BGLD	BGLD	8	TC, TG, HDL-C
Zhang and Chen 2010 [19]	22/23	XZK + BGLD	BGLD	12	TC, TG, HDL-C, LDL-C
Yang et al. 2013 [20]	32/34	XZK + BGLD	BGLD	12	TC, TG, HDL-C, LDL-C
Tongcui and Bihui 2002 [21]	35/30	XZK + BGLD	BGLD	8	TC, TG, HDL-C, LDL-C
	35/30	XZK + BGLD	BGLD	12	TC, TG, HDL-C, LDL-C
Lin et al. 2000 [22]	48/32	XZK + BGLD	Placebo + BGLD	8	TC, TG, HDL-C, Effectiveness
Chen et al. 2000 [23]	30/30	XZK + BGLD	Placebo + BGLD	8	TC, TG, HDL-C, Effectiveness
Chen 2004 [24]	135/133	XZK + BGLD	Placebo + BGLD	12	TC, TG, HDL-C, LDL-C
Hao 2002 [25]	52/53	XZK + BGLD	Duoxikang + BGLD	8	TC, TG, HDL-C, Effectiveness

**Table 2:** The result of the continuous variables.

Study	Intervention	Dosage	Treatment	TC	TG	HDL-C	LDL-C
Liao and Chen 2010 [7]	XZK + BGLD	0.6 g/time bid	Before	6.98 ± 0.67	2.02 ± 0.35	1.05 ± 0.11	3.92 ± 0.42
			After	4.45 ± 0.43	1.67 ± 0.40	1.24 ± 0.19	2.67 ± 0.48
	Simvastatin + BGLD	20 mg/time qd	Before	6.85 ± 0.52	2.31 ± 0.36	1.06 ± 0.13	3.76 ± 0.24
			After	4.65 ± 0.42	1.91 ± 0.62	1.17 ± 0.13	2.75 ± 0.42

Liu and Dong 2008 [8]	XZK + BGLD	0.6 g/time bid	Before	5.97 ± 0.38	2.53 ± 0.35	1.04 ± 0.12	3.94 ± 0.43
			After	4.46 ± 0.42	1.65 ± 0.39	1.23 ± 0.18	2.67 ± 0.47
	Simvastatin + BGLD	10 mg/time qd	Before	6.03 ± 0.45	2.49 ± 0.36	1.09 ± 0.16	3.78 ± 0.46
			After	4.68 ± 0.43	1.84 ± 0.51	1.16 ± 0.14	2.75 ± 0.43

Wang et al. 2005 [9]	XZK + BGLD	0.6 g/time bid	Before	6.8 ± 1.9	3.4 ± 1.2	1.2 ± 0.5	3.9 ± 1.5
			After	4.5 ± 1.6	2.2 ± 0.9	1.4 ± 0.6	2.5 ± 1.3
	Pravastatin + BGLD	20 mg/d	Before	6.7 ± 1.2	3.3 ± 1.1	1.1 ± 0.4	4.1 ± 1.1
			After	4.4 ± 1.0	2.1 ± 0.8	1.3 ± 0.7	2.6 ± 1.5
	XZK + BGLD	0.6 g/time bid	Before	6.8 ± 1.9	3.4 ± 1.2	1.2 ± 0.5	3.9 ± 1.5
			After	4.5 ± 1.6	2.2 ± 0.9	1.4 ± 0.6	2.5 ± 1.3
	Fenofibrate + BGLD	0.2 g/time qd	Before	6.8 ± 0.9	3.3 ± 0.9	1.2 ± 0.6	3.9 ± 1.2
			After	5.2 ± 1.2	1.7 ± 0.7	1.5 ± 0.8	3.2 ± 1.4

Lanfan and Zurong 2005 [10]	XZK + BGLD	0.6 g/time bid	Before	6.86 ± 0.62	3.49 ± 1.15	0.88 ± 0.14	4.15 ± 1.05
			After	4.81 ± 1.18	2.18 ± 0.55	1.13 ± 0.22	2.88 ± 1.01
	Simvastatin + BGLD	10–20/time qd	Before	6.91 ± 0.68	3.32 ± 1.16	0.87 ± 0.13	4.13 ± 1.02
			After	4.76 ± 1.19	2.15 ± 0.56	1.06 ± 0.21	2.69 ± 1.03

Tong 2012 [11]	XZK + BGLD	0.6 g/time bid	Before	6.24 ± 1.52	3.11 ± 1.28	1.06 ± 0.24	4.32 ± 1.27
			After	4.54 ± 1.14	2.11 ± 1.24	1.65 ± 0.28	3.11 ± 1.13
	Simvastatin + BGLD	20/time qd	Before	6.26 ± 1.50	3.14 ± 1.61	1.06 ± 0.38	4.36 ± 1.22
			After	4.95 ± 1.46	2.71 ± 1.20	1.62 ± 0.24	2.45 ± 1.07

Zhe 2012 [12]	XZK + BGLD	0.6 g/time bid	Before	6.55 ± 1.09	2.11 ± 0.34	1.05 ± 0.11	3.89 ± 0.43
			After	4.53 ± 0.49	1.42 ± 0.21	1.34 ± 0.15	2.56 ± 0.51
	Atorvastatin + BGLD	10 mg/time qd	Before	6.78 ± 0.79	2.30 ± 0.41	1.01 ± 0.14	3.77 ± 0.35
			After	4.87 ± 0.55	1.96 ± 0.42	1.12 ± 0.09	2.93 ± 0.28

Xueling 2007 [13]	XZK + BGLD	0.6 g/time bid	Before	6.79 ± 1.50	3.38 ± 1.52	1.01 ± 0.22	4.20 ± 1.30
			After	5.51 ± 1.22	2.12 ± 1.27	1.98 ± 0.38	2.65 ± 1.10
	Fluvastatin + BGLD	40 mg/time qd	Before	6.82 ± 1.54	3.41 ± 1.82	1.07 ± 0.26	4.25 ± 1.20
			After	5.45 ± 1.42	2.42 ± 1.10	2.24 ± 0.59	2.20 ± 1.18

Chen 2013 [14]	XZK + BGLD	0.6 g/time bid	Before	5.90 ± 1.12	2.81 ± 1.21	0.98 ± 0.43	3.85 ± 0.86
			After	3.93 ± 0.89	1.89 ± 1.13	1.39 ± 0.51	2.6 ± 0.78
	Simvastatin + BGLD	20 mg/time qd	Before	5.87 ± 1.38	2.37 ± 0.98	1.01 ± 0.35	3.73 ± 0.67
			After	4.00 ± 0.93	1.90 ± 1.15	1.37 ± 0.54	2.47 ± 0.93

Yang 2008 [15]	XZK + BGLD	0.6 g/time bid	Before	6.23 ± 1.51	3.12 ± 1.42	1.07 ± 0.22	4.36 ± 1.22
			After	4.53 ± 1.12	2.12 ± 1.20	1.63 ± 0.31	3.10 ± 1.12
	Simvastatin + BGLD	20 mg/time qd	Before	6.27 ± 1.49	3.13 ± 1.62	1.08 ± 0.35	4.37 ± 1.21
			After	4.94 ± 1.45	2.72 ± 1.19	1.59 ± 0.28	2.47 ± 1.03

Jianhui 2013 [16]	XZK + BGLD	0.6 g/time bid	Before	6.7 ± 1.3	2.2 ± 0.4	1.1 ± 0.2	3.9 ± 0.7
			After	4.6 ± 0.7	1.4 ± 0.3	1.4 ± 0.1	2.7 ± 0.3
	Rosuvastatin + BGLD	10 mg/time qd	Before	6.9 ± 1.0	2.3 ± 0.6	1.0 ± 0.1	3.8 ± 0.5
			After	5.1 ± 0.4	1.9 ± 0.4	1.1 ± 0.2	3.0 ± 0.3

Hui and Zhaoliang 2005 [17]	XZK + BGLD	0.6 g/time bid	Before	6.2 ± 1.5	3.15 ± 1.52	1.06 ± 0.23	4.53 ± 1.23
			After	4.5 ± 1.2	2.16 ± 1.25	1.62 ± 0.38	3.09 ± 1.15
	Simvastatin + BGLD	20 mg/time qd	Before	6.3 ± 1.5	3.16 ± 1.82	1.08 ± 0.36	4.38 ± 1.19
			After	4.9 ± 1.8	2.71 ± 1.09	1.19 ± 0.27	2.48 ± 1.02

Zhang and Chen 2010 [19]	XZK + BGLD		Before	6.55 ± 0.32	2.81 ± 0.46	1.15 ± 1.4	3.85 ± 0.71
		0.6 g/time bid	After	5.13 ± 0.51	1.54 ± 0.38	1.4 ± 0.25	2.75 ± 0.29
	BGLD		Before	6.71 ± 0.41	2.96 ± 0.55	1.17 ± 0.31	3.93 ± 0.82
			After	6.45 ± 0.73	2.87 ± 0.49	1.18 ± 0.78	3.91 ± 0.97

Tang and Weng 2002 [18]	XZK + BGLD		Before	6.06 ± 2.35	3.14 ± 1.82	0.95 ± 0.42	
		0.6 g/time bid	After	4.89 ± 1.81	1.71 ± 0.73	1.3 ± 0.45	
	BGLD		Before	6.1 ± 2.62	3.3 ± 0.9	0.91 ± 0.27	
			After	6.06 ± 2.35	3.2 ± 0.5	0.95 ± 0.42	

Yang et al. 2013 [20]	XZK + BGLD		Before	5.86 ± 1.14	2.6 ± 1.01	0.94 ± 0.2	3.39 ± 1.22
		0.6 g/time bid	After	5 ± 0.68	1.73 ± 0.92	1.1 ± 0.17	2.56 ± 0.91
	BGLD		Before	5.81 ± 0.75	2.64 ± 1.12	0.97 ± 0.24	2.92 ± 0.57
			After	5.63 ± 0.91	2.49 ± 0.86	1.05 ± 0.19	2.8 ± 0.35

Tongcui and Bihui 2002 [21]	XZK + BGLD		Before	6.6 ± 0.3	3.1 ± 0.85	1.2 ± 0.1	3.7 ± 0.3
		0.6 g/time bid	After	5.6 ± 0.3	1.6 ± 0.2	1.4 ± 0.2	2.6 ± 0.3
	BGLD		Before	6.4 ± 0.4	2.9 ± 0.4	1.1 ± 0.1	3.8 ± 0.2
			After	6.4 ± 0.3	2.1 ± 0.5	1.2 ± 0.1	3.7 ± 0.1

Tongcui and Bihui 2002 [21]	XZK + BGLD		Before	6.6 ± 0.3	3.1 ± 0.85	1.2 ± 0.1	3.7 ± 0.3
		0.6 g/time bid	After	5.3 ± 0.2	1.4 ± 0.3	1.6 ± 0.1	2.4 ± 0.3
	BGLD		Before	6.4 ± 0.4	2.9 ± 0.4	1.1 ± 0.1	3.8 ± 0.2
			After	6.4 ± 0.3	2.1 ± 0.4	1.3 ± 0.2	3.5 ± 0.3

Chen 2004 [24]	XZK + BGLD		Before	8.12 ± 0.81	2.16 ± 0.98	1.03 ± 0.52	4.46 ± 0.93
		0.6 g/time bid	After	4.51 ± 0.95	1.18 ± 0.83	2.42 ± 0.56	2.50 ± 0.89
	Placebo + BGLD		Before	7.98 ± 0.87	2.14 ± 0.86	1.06 ± 0.49	4.48 ± 0.95
			After	7.55 ± 0.88	2.17 ± 0.98	1.08 ± 0.53	4.39 ± 0.97

**Table 3:** The result of the dichotomous variables.

Study	Intervention	TC		TG		HDL-C	
		Total effect	No effect	Total effect	No effect	Total effect	No effect
Lin et al. 2000 [22]	XZK + BGLD	39/41	2/41	41/43	2/43	9/10	1/10
	Placebo + BGLD	12/19	7/19	10/22	12/22	2/6	4/6

Chen et al. 2000 [23]	XZK + BGLD	23/25	2/25	21/23	2/23	7/8	1/8
	Placebo + BGLD	11/21	10/21	9/26	17/26	1/7	6/7

Hao 2002 [25]	XZK + BGLD	41/46	5/46	38/44	6/44	27/45	28/45
	DXK + BGLD	21/41	20/41	29/48	19/48	8/41	13/41
